# Donor Lymphocyte Infusions After Allogeneic Stem Cell Transplantation in Acute Leukemia: A Survey From the Gruppo Italiano Trapianto Midollo Osseo (GITMO)

**DOI:** 10.3389/fonc.2020.572918

**Published:** 2020-10-15

**Authors:** Francesca Patriarca, Alessandra Sperotto, Francesca Lorentino, Elena Oldani, Sonia Mammoliti, Miriam Isola, Alessandra Picardi, William Arcese, Giorgia Saporiti, Roberto Sorasio, Nicola Mordini, Irene Cavattoni, Maurizio Musso, Carlo Borghero, Caterina Micò, Renato Fanin, Benedetto Bruno, Fabio Ciceri, Francesca Bonifazi

**Affiliations:** ^1^Clinica Ematologica e Centro Trapianti, Azienda Sanitaria Universitaria Friuli Centrale, Udine, Italy; ^2^Department of Medical Area (DAME) Università di Udine, Udine, Italy; ^3^Unitá Operativa Complessa (UOC) Ematologia e Trapianto, Istituto di Ricovero e Cura a carattere scientifico (IRCSS) Ospedale San Raffaele, Milan, Italy; ^4^Unitá Operativa Complessa (UOC) Ematologia e Trapianto, Azienda Socio Sanitaria Territoriale (ASST) Papa Giovanni XXIII, Bergamo, Italy; ^5^Trial Office Gruppo Trapianto Di Midollo Osseo e Terapie Cellulari (GITMO), Genova, Italy; ^6^Istituto di Statistica, Department of Medical Area (DAME) Università di Udine, Udine, Italy; ^7^Unitá Operativa Complessa (UOC) Ematologia con Trapianto, Azienda Ospedaliera (AO) Cardarelli, Naples, Italy; ^8^Unitá Operativa Complessa (UOC) Ematologia, Azienda Ospedaliera Universitaria (AOU) Policlinico Tor Vergata, Rome, Italy; ^9^Ematologia-Centro Trapianti di Midollo-Fondazione Istituto di Ricovero e Cura a carattere scientifico (IRCSS) Ca' Granda Ospedale Maggiore Policlinico, Milan, Italy; ^10^Unitá Operativa Complessa (UOC) Ematologia, Azienda Ospedaliera (AO) S. Croce, Cuneo, Italy; ^11^UOC Ematologia, Azienda Ospedaliera (AO) di Bolzano, Genova, Italy; ^12^Unitá Operativa Onco-ematologia e (UO) Oncoematologia e Trapianto Midollo Osseo (TMO), Ospedale LaMaddalena, Palermo, Italy; ^13^Unitá Operativa Complessa (UOC) Ematologia, Ospedale San Bortolo, Vicenza, Italy; ^14^Unitá Operativa Complessa (UOC) Ematologia, Azienda Ospedaliera Universitaria (AOU) Città della Salute e della Scienza, Turin, Italy; ^15^Ematologia, Azienda Ospedaliera-Universitaria di Bologna, Bologna, Italy

**Keywords:** donor lymphocyte infusions, relapse, allogeneic stem cell transplantation, acute leukemia, pre-emptive treatment

## Abstract

We conducted a retrospective multicenter study including pediatric and adult patients with acute leukemia (AL) who received donor lymphocyte infusions (DLIs) after allogeneic hematopoietic stem cell transplantation (HCT) between January 1, 2010 and December 31, 2015, in order to determine the efficacy and toxicity of the immune treatment. Two hundred fifty-two patients, median age 45.1 years (1.6–73.4), were enrolled from 34 Italian transplant centers. The underlying disease was acute myeloid leukemia in 180 cases (71%). Donors were HLA identical or 1 locus mismatched sibling (40%), unrelated (40%), or haploidentical (20%). The first DLI was administered at a median time of 258 days (55–3,784) after HCT. The main indication for DLI was leukemia relapse (73%), followed by mixed chimerism (17%), and pre-emptive/prophylactic use (10%). Ninety-six patients (38%) received one single infusion, whereas 65 (26%), 42 (17%), and 49 patients (19%) received 2, 3, or ≥4 infusions, respectively, with a median of 31 days between two subsequent DLIs. Forty percent of evaluable patients received no treatment before the first DLI, whereas radiotherapy, conventional chemotherapy or targeted treatments were administered in 3, 39, and 18%, respectively. In informative patients, a few severe adverse events were reported: grade III–IV graft versus host disease (GVHD) (3%), grade III–IV hematological toxicity (11%), and DLI-related mortality (9%). Forty-six patients (18%) received a second HCT after a median of 232 days (32–1,390) from the first DLI. With a median follow-up of 461 days (2–3,255) after the first DLI, 1-, 3-, and 5- year overall survival (OS) of the whole group from start of DLI treatment was 55, 39, and 33%, respectively. In multivariate analysis, older recipient age, and transplants from haploidentical donors significantly reduced OS, whereas DLI for mixed chimerism or as pre-emptive/prophylactic treatment compared to DLI for AL relapse and a schedule including more than one DLI significantly prolonged OS. This GITMO survey confirms that DLI administration in absence of overt hematological relapse and multiple infusions are associated with a favorable outcome in AL patients. DLI from haploidentical donors had a poor outcome and may represent an area of further investigation.

## Introduction

Disease relapse is the leading cause of treatment failure and mortality in patients with acute leukemia (AL) undergoing allogeneic hematopoietic stem cell transplantation (HCT). Most relapses occur within 1 year after HCT and exhibit a progressive clinical course. Two main mechanisms may be responsible for relapse after HCT: tumor cells may escape from the impact of pre-transplant conditioning chemotherapy regimens or tumor cells may evade post-transplant immune control. Many treatment strategies, including pharmaceutical and cell-based treatments, have been developed and tested to prevent and treat relapses. Donor lymphocyte infusion (DLI) is a form of adoptive immunotherapy aiming to enhance the graft-versus-leukemia (GVL) effect after HCT. DLIs were first used in patients suffering from chronic myeloid leukemia (CML) relapse after HCT. In these patients, especially in the case of cytogenetic or molecular relapse, DLIs achieved a high rate of complete responses (up to 80%) compared to patients with other hematological disorders (1–3). In patients with acute leukemia, clinical responses have been reported to be fewer, particularly in the case of overt relapse and in the presence of acute lymphoid leukemia (4–8). Moreover, clinical success is limited by the occurrence of acute and chronic graft-versus-host disease (GVHD), marrow aplasia and infections, which can be all causes of treatment-related mortality in up to 20% of patients (9, 10). To determine the efficacy and toxicity of DLIs and to identify potential factors influencing the outcome, we conducted a retrospective multicenter study including patients with AL who received DLIs after HCT from related and unrelated donors.

## Methods

### Study Design and Information Collection

This was a multicenter retrospective study carried out in Italian transplant centers coordinated by the Gruppo Italiano per il Trapianto Midollo Osseo e Terapia Cellulare (GITMO) network. Criteria for patient eligibility were the following: adult and pediatric patients, without age limit; diagnosis of acute myeloid leukemia (AML) or acute lymphoblastic leukemia (ALL); any stage of disease at transplant; first HCT from HLA-identical sibling or volunteer or mismatched related donor; myeloablative or reduced-intensity conditioning (RIC) regimen; and at least 1 unmanipulated DLI administered between January 1, 2010 and December 31, 2015. Exclusion criteria included: DLI treatment after second or further HCT, T-cell depleted transplant, and diagnosis other than AL. The primary endpoint was overall survival (OS). The secondary endpoints were: indications for DLI administration and the DLI schedule most commonly adopted among the GITMO centers, response rate, non-relapse mortality (NRM), hematological toxicity, and acute and chronic GVHD incidence. Thirty-four GITMO centers accredited for allogeneic HCT participated in the study. Information was collected in two phases. In the first phase, a survey was conducted to collect the data of the 34 participating centers from the GITMO registry. The data collected were the following: demographic data, relationship and HLA compatibility of patients and donors, AL type, conditioning, stem cell source, GVHD prophylaxis, acute and chronic GVHD after transplantation, relapse, patient and disease status at last follow-up, date of DLI administration, clinical indication for DLI administration, possible treatments administered before DLI, and date of possible second HCT. In the second phase, the participating centers were asked to provide data that were missing from the GITMO registry. The data provided were the following: cell doses, transfusion schedule, hematological and non-hematological toxicity, acute and chronic GVHD after DLIs, and disease response. Fifteen centers agreed to and completed the second part of the study.

### Ethics Section

The study involving human participants was reviewed and approved by the Ethics Committee of the center of the national principal investigator, called “Comitato Unico Regionale Friuli-Venezia-Giulia” on 2017, 3 October (Protocol Number 26522) and by the Ethics Committees of all participating institutions. A written and informed consent was obtained from all patients according to the Declaration of Helsinki.

### Definitions

DLI was defined as transfusion of unstimulated lymphocyte concentrates, collected from the original stem cell donor as buffy coat preparations, or as transfusion of unmanipulated mobilized peripheral blood stem cells (PBSC). RIC regimens were defined as described by Bacigalupo et al. (11). Acute GVHD was graded according to the 1994 Consensus Conference on acute GVHD grading criteria (12), and chronic GVHD was staged according to the criteria developed by the National Institute of Health (13). Hematological relapse was defined by recurrence of blasts in PB or infiltration of bone marrow (BM) by more than 5% blasts. Pre-emptive treatment was defined as DLI administration in cases of reappearance of minimal residual disease (MRD) (any AL cytogenetic or molecular or phenotypic marker previously detected at diagnosis) in absence of hematological relapse. Prophylaxis was defined as DLI treatment to prevent hematological relapse in patients with negative MRD. Mixed chimerism was defined as failure to achieve >95% of donor cells or decreased chimerism, with evidence of AL complete remission. Targeted therapy before DLI included hypomethylating agents in patients with AML and tyrosine kinase inhibitors in patients with ALL.

### Statistical Analysis

Data were collected in an XLS database and imported into Stata/SE 9.0 for Windows (StataCorp, College Station, TX) for statistical analysis. The close-out date for analysis was December 2018. The starting points of our analyses were day of first HCT and day of first DLI. NRM was defined as death due to all causes not related to leukemia and was estimated with the cumulative incidence method. OS was defined as the time (days) from the aforementioned starting points to either death or last observation and was described using the Kaplan-Meier approach.

In univariate analysis, variables considered as possible prognostic factors were: age at transplantation (years), sex, AL type (AML or ALL), conditioning intensity (myeloablative or RIC), GVHD prophylaxis (calcineurin inhibitors plus methotrexate or calcineurin inhibitors plus methotrexate plus antithymocyte serum or post-transplant cyclophosphamide or other platforms), donor type (HLA-identical plus 1 antigen mismatched related donor vs. unrelated donor vs. haploidentical donor), time between HCT and first DLI (≤180 days, 181–365 days, 366–730 days, >730 days), indication for DLI administration (relapse or mixed chimerism or pre-emptive treatment/prophylaxis), treatment administered before DLI (no pharmacological treatment or conventional chemotherapy or targeted therapy), number of infusions, and acute or chronic GVHD after HCT (yes or no). Acute and chronic GVHD were treated as time-dependent variables. Multivariate stepwise analyses included all variables found to be significant at *p* ≤ 0.10 on univariate analysis. Retention in the stepwise model required the variable to be significant at *p* ≤ 0.05.

## Results

### Patient and Transplant Information (Table 1)

Two hundred fifty-two patients were enrolled from 34 Italian transplant centers. Thirty centers (86%) provided data for <10 patients. One hundred thirty-three patients (53%) were male and median age at transplant was 45.1 years (range 1.6–73.4). Only 13 patients (5%) were younger than 18 years. The underlying disease was AML in 180 patients (71%), ALL in 68 patients (27%), and biphenotypic AL in 4 patients (2%). The majority of HCTs (180, 71%) were performed between 2011 and 2015, whereas the other procedures were done before 2011. Twenty percent of patients had active AL at transplant. Preparative regimens before HCT were myeloablative in 179 transplants (72%). One hundred fifty patients (60%) had a related donor, who was HLA-identical sibling, 1 locus HLA mismatched, or haploidentical in 98, 3, and 49 cases, respectively; 102 patients (40%) had an unrelated donor. An high resolution DNA typing was performed at HLA-A, -B, -C, -DRB1 loci; 65 out of 91 evalutable unrelated transplants (71%) were HLA-matched, while a single mismatch at HLA-A, -B, or -C locus was present in 10 (11%), 6 (7%), and 10 recipient and donor pairs (11%), respectively. One hundred sixty-nine patients (67%) received PBSC, and 83 (33%) received BM. GVHD prophylaxis consisted of calcineurin inhibitor (cyclosporine or tacrolimus) plus methotrexate in 80 patients (32%), calcineurin inhibitor plus methotrexate plus antithymocyte globulin (ATG) in 120 patients (48%), post-transplant cyclophosphamide-based prophylaxis in 13 patients (5%), and other platforms in the remaining 39 patients (15%). Most common miscellaneous GVHD prophylaxis regimens were used in haploidentical transplants and included rapamycin-based and ATG plus basiliximab-based platforms. Sixty-nine of the evaluable patients (32%) developed grade I-IV acute GVHD, which reached grade III–IV only in 10 cases (4%). Chronic GVHD occurred in 98 of evaluable patients (47%) and was severe in 26 cases (12%).

**Table 1:** Characteristics of patients and allogeneic transplants.

Total number of patients	252
Sex: male	133 (53%)
Median age (range) at transplant	45.1 (1.6–73.4)
Age <18 years	13 (5%)
**Diagnosis**	
AML [secondary AML]	180 (71%) [32 (17%)]
ALL [Ph+ ALL]	68 (27%) [8 (12%)]
Biphenotypic AL	4 (2%)
**Transplant date**	
≤2005	3 (2%)
2006–2010	69 (27%)
2011–2015	180 (71%)
**Disease status at transplant**	
Not treated	2 (1%)
1 CR	135 (54%)
≥2 CR	61 (25%)
Primary induction	26 (10%)
Relapse	26 (10%)
Missing	2
**Donor**	
HLA-matched sibling	98 (39%)
1 locus mismatched related	3 (1%)
Haploidentical	49 (20%)
Unrelated	102 (40%)
**Stem cell source**	
Bone marrow	83 (33%)
Peripheral blood	169 (67%)
**Conditioning regimen**	
Myeloablative (with TBI)	42 (17%)
Myeloablative (only drugs)	137 (55%)
Reduced intensity	71 (28%)
**GVHD prophylaxis**	
CyA/FK + MTX	80 (32%)
Cya/FK + MTX + ATG	120 (48%)
PT-CY	13 (5%)
Other	36 (15%)
Missing	3
**Acute GVHD**	
Grade 0	169 (68%)
Grade I	42 (17%)
Grade II	27 (11%)
Grade III–IV	10 (4%)
Missing	4
**Chronic GVHD**	
Absent	109 (53%)
Mild-moderate	72 (35%)
Severe	26 (12%)
Missing	45

*No, number; AML, acute myeloid leukemia; ALL, acute lymphoblastic leukemia; Ph+, Philadelphia chromosome positive; CR, complete remission; TBI, total body irradiation; CyA, cyclosporine; FK, tacrolimus; MTX, methotrexate; ATG, antithymocyte globulin; PT-Cy, post-transplant cyclophosphamide*.

### DLI Administration (Table 2)

All patients received at least one DLI. The first DLI was administered at a median time of 258 days (55–3,784) after HCT. The main indication for DLI was leukemia relapse after HCT (172 patients, 73%), followed by mixed chimerism (39 patients, 17%) and pre-emptive/prophylactic use (24 patients, 10%). Ninety-six patients out of 252 (38%) received one single infusion, whereas 65 (26%), 42 (17%), and 49 patients (19%) received 2, 3, or ≥4 infusions, respectively, with a median of 31 days between two subsequent DLIs. Forty percent of evaluable patients received no treatment before the first DLI, whereas radiotherapy, conventional chemotherapy or targeted treatments were administered in 3, 39, and 18%, respectively. The percentage of patients who did not receive any treatment in association with DLIs increased to 87 and 90% after the second and third DLI, respectively. The median dose of the first DLI was 1 × 10^6^/kg (0.01–10) for the informative patients. In case of multiple infusions, an escalating schedule was mainly chosen, with median doses ranging from 1 × 10^6^/kg CD3+ lymphocytes (0.01–10) for the first infusion to 10 × 10^6^/kg CD3+ lymphocytes (0.05–50) for the fifth or further infusion. Median and range of CD3+ cells/kg of the first DLI were 1 × 10^6^ (0.5–10), 1 × 10^6^ (0.1–10), and 0.3 × 10^6^ (0.05–1) in recipients of DLIs from HLA identical sibling, unrelated and haploidentical donors, respectively. A sequential schedule was administered to 36/98 (37%) recipients of DLIs from HLA identical sibling donors, 32/102 (31%) recipients of DLIs from unrelated donors and to 25/52 (48%) recipients of DLIs from haploidentical donors, respectively (*p* = 0.127) After the first DLI, acute GVHD was reported in 13% of informative patients and was grade III-IV in 3% of patients. The percentage of patients who developed acute GVHD decreased to 11 and 7% after the second and third DLI, respectively. In contrast, the percentage of evaluable patients who developed chronic moderate-severe GVHD requiring treatment increased from 2% after the first DLI to 7 and 14% after the second and third DLI, respectively. Grade III-IV neutropenia and/or thrombocytopenia occurred in 11% of the evaluable patients after the first DLI and the rate was not significantly different after subsequent infusions. Severe infections were reported in 6 out 98 informative DLIs (6%) and included invasive mycoses (2 patients), viral infections (2 cases), and recurrent bacterial enteritis (2 patients). Forty-five patients who received DLI because of relapse were evaluable for response after cell infusion: 14 patients (31%) reached complete remission, 16 patients (35%) had stable disease, and 15 (33%) experienced leukemia progression. Forty-six patients (18%) received a second HCT after a median of 922 days (149–1,970) from the first HCT and after a median of 232 days (32–1,390) from the first DLI. There was no significant difference in the proportion of patients undergoing second HCT after receiving DLI according to immunotherapy indication. In fact, 35 out of 172 patients (20%) who received DLI because of relapse required a second transplant compared to 3 out of 39 (8%) and 3 out of 24 (12%) of those who were treated with DLI because of mixed chimerism or as prevention, respectively (*p* = 0.136).

**Table 2:** Characteristics of DLIs.

	**1stDLI**	**2ndDLI**	**3rdDLI**	**4thDLI**	**≥5thDLI**
**Total number of cases**	252	156	91	49	53
**Time between transplant and first DLI (days)**	258 (55–3,784)				
**Time between DLIs**		29 (1-1015)	30 (2-636)	33 (7-623)	31 (13-441)
**DLI indication**					
AL relapse	172/235 (73%)	–	–	–	–
Mixed chimerism	39/235 (17%)				
Pre-emptive/prophylaxis	24/235 (10%)				
Missing	17				
**Treatment before DLIs**					
No treatment	41/103 (40%)	110/126 (87%)	70/78 (90%)	40/47 (85%)	28/46 (61%)
Radiotherapy	3/103 (3%)	–	1/78 (1%)	–	1/46 (2%)
Chemotherapy	40/103 (39%)	5/126 (4%)	2/78 (2%)	2/47 (5%)	–
Targeted therapy	19/103 (18%)	11/126 (9%)	6/78 (7%)	7/47 (15%)	17/46 (37%)
Missing	149	30	13	2	7
**Dose (× 10^6^/kg)**					
Median (range)	1 (0.01–10)	2 (0.01–64)	5 (0.05–100)	10 (0.05–50)	10 (0.05–50)
Missing	198	126	70	33	38
**Acute GVHD**					
Grade 0	141/163 (87%)	94/106 (89%)	64/69 (93%)	36/39 (82%)	43/48 (90%)
Grade I–II	17/163 (10%)	9/106 (8%)	3/69 (4%)	2/39 (5%)	4/48 (8%)
Grade III–IV	5/163 (3%)	3/106 (3%)	2/69 (3%)	1/39 (3%)	1/48 (2%)
Missing	89	50	22	10	
**Grade IV hematological toxicity**					
Number of cases	9/82 (11%)	4/35 (11%)	4/26 (15%)	2/18 (11%)	–
Missing	170	121	65	31	53

*AL, acute leukemia*.

### Outcome

**Figure 1:**
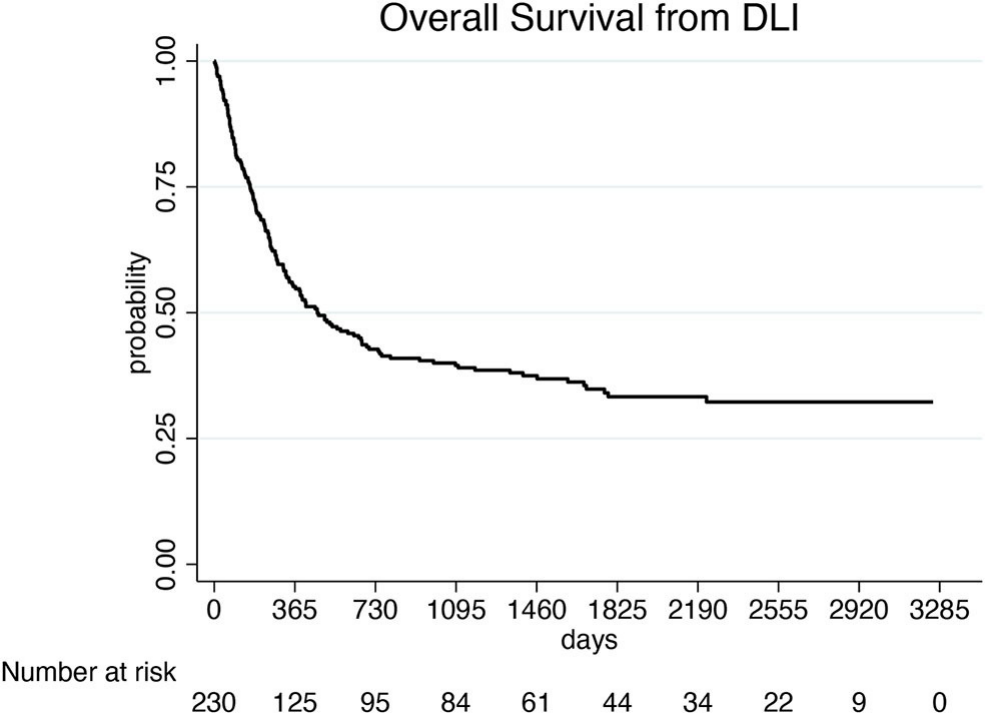
Overall survival of the 252 patients treated with DLIs.

**Figure 2:**
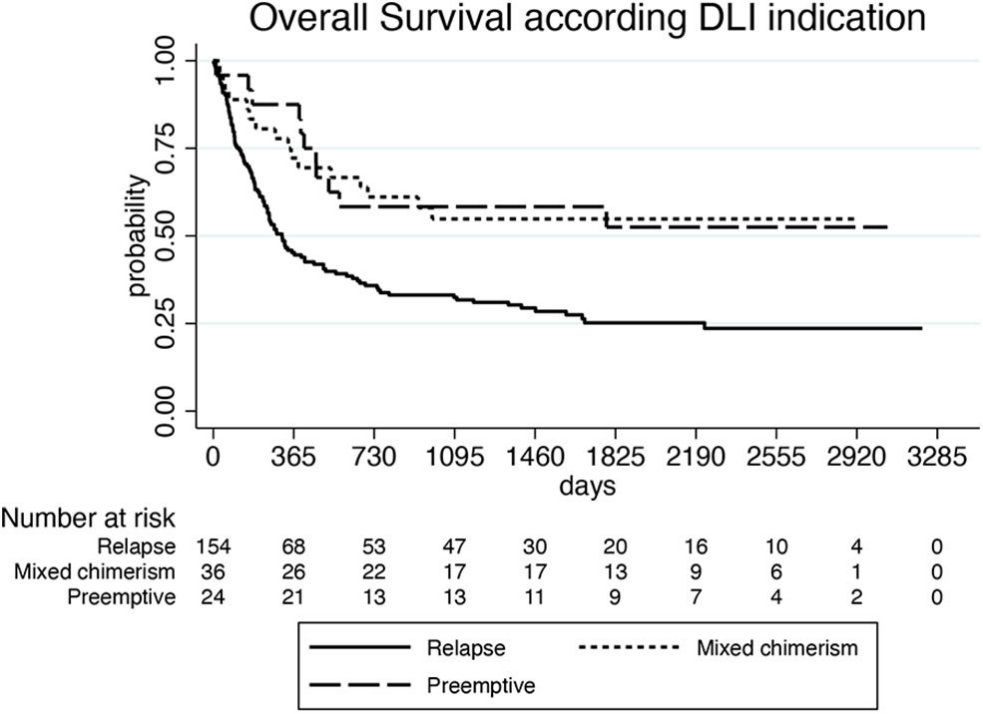
Overall survival according to DLI indication.

**Figure 3:**
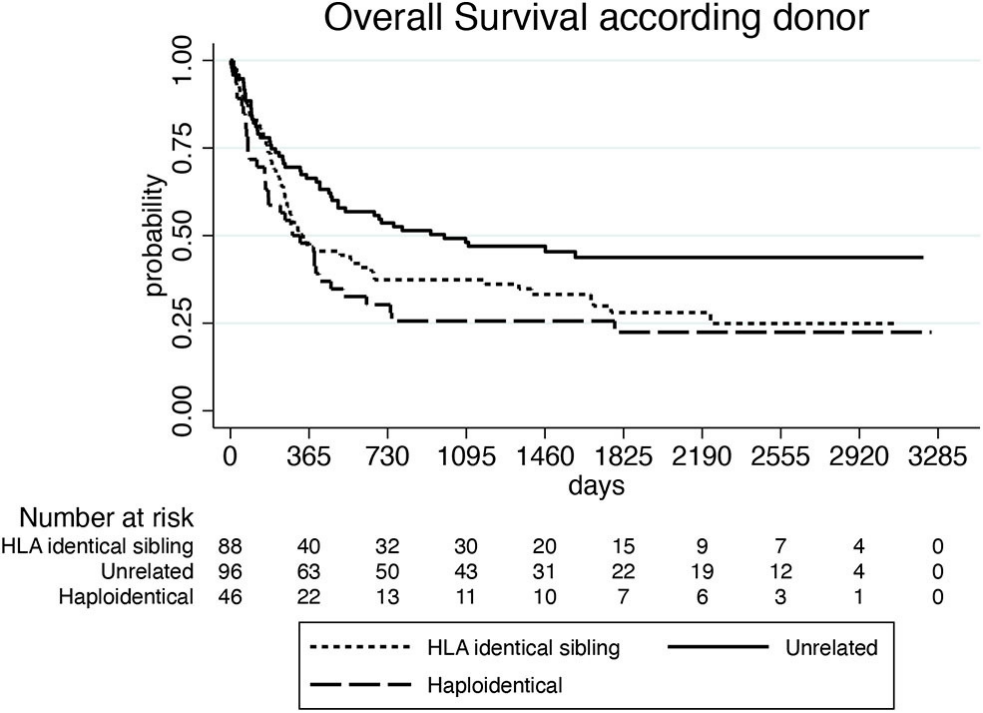
Overall survival according to donor.

**Figure 4:**
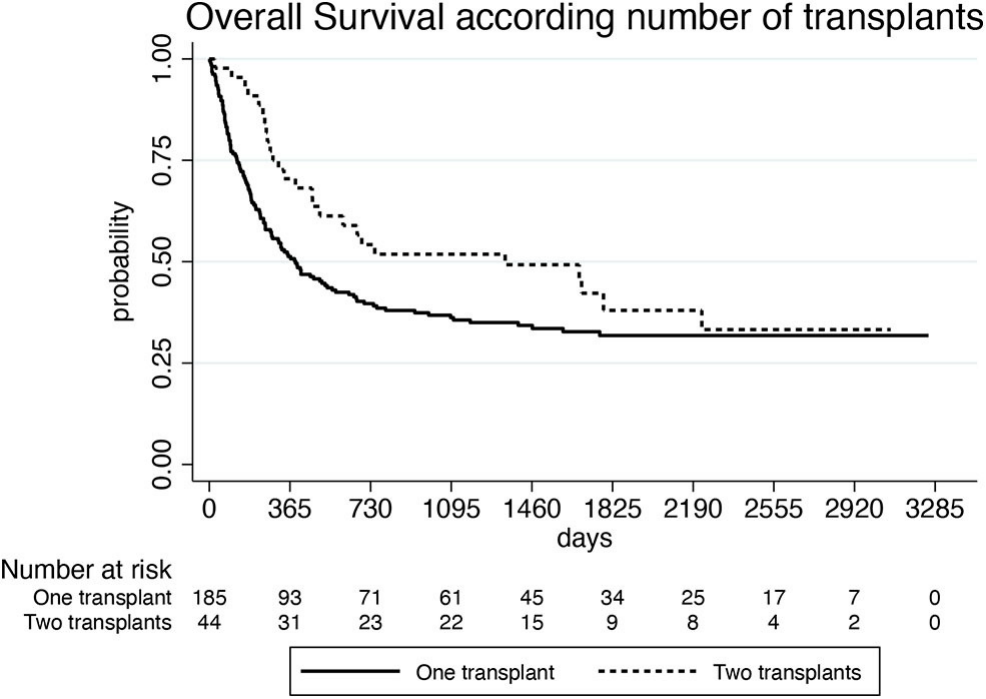
Overall survival according to number of allogeneic transplants.

With a median follow-up of 878 days (55–6,754) after the first HCT and 461 days (2–3,255) after the first DLI, 81 of the 248 evaluable patients (33%) were alive and 167 (67%) were dead. Of these latter, 141 (84%) died because of leukemia progression and 26 (16%) because of NRM. Causes of NRM were related to DLI (15 patients, 9%), second HCT (6 patients, 4%), secondary malignancy (2 cases, 1%), and to other causes (3 patients, 2%). NRM events were equally distributed between patients treated in small centers (providing data of ≤10 patients) and large centers (providing data of more than 10 patients): in fact, 12 out of 127 patients (9%) from small centers and 14 out of 121 patients (11%) from large centers died because of NRM (*p* = 0.736*)*. Median survival was 915 days (55–6,754) from the first HCT and 466 days (2–3,255) from the first DLI, respectively. One-, three-, and five-year OS of the whole group from the beginning of DLI treatment was 55, 39, and 33%, respectively (Figure 1). Prognostic factors that were significantly (*p* < 0.10) associated with OS after DLI in the univariate proportional hazards model were: age, donor type, treatment before DLI, indication for DLI, number of DLI, time between transplant and first DLI (Table 3). In multivariate analysis, older recipient age and transplants from haploidentical donors significantly reduced OS (HR 1.020; 95% CI 1.008–1.033; *p* = 0.001 and HR 2.815; 95% CI 1.702–4.656; *p* = 0.000, respectively), whereas DLI for mixed chimerism or as pre-emptive/prophylactic treatment compared to AL relapse and a schedule including more than one DLI significantly prolonged OS (HR 0.379; 95% CI 0.219–0.646; *p* = 0.000; HR 0.202; 95% CI 0.098–0.415; *p* = 0.000; HR 0.876; 95% CI 0.767–1.000; *p* = 0.050, respectively). Moreover, a time between transplant and first DLI longer than 2 years significantly improved OS (HR 0.411; 95% CI 0.229–0.740; *p* = 0.003; Table 4). Patients who received DLI because of relapse reported a 3-year OS of 32%, which was significantly lower than the 3-year OS of 55 and 58% for those patients who were treated with DLI because of mixed chimerism (*p* = 0.002) or pre-emptive/prophylactic use (*p* = 0.008; Figure 2). Moreover, transplants from haploidentical donors showed a 3-year OS of 25%, which was significantly lower than that reported in transplants from unrelated donors (3-year OS 48%, *p* = 0.000; Figure 3). In addition, patients who received a second HCT after receiving DLI showed a trend of longer OS compared to patients who received only one transplant followed by DLI (*p* = 0.077; Figure 4).

**Table 3:** Univariate analysis of overall survival data from first DLI.

**Factor**	**HR**	**95%CI**	***p***
**Age**			
Modeled as continuous variable	1.012	1.001–1.024	**0.027**
**Sex**			
Male	1	0.683–1.403	0.623
Female	1.084		
**Diagnosis**			
AML	1	0.742–1.495	0.907
ALL	0.979		
**Donor**			
Unrelated	1		
HLA-identical sibling or 1 locus mismatched related	1.494	1.031–2.165	**0.034**
Haploidentical	1.843	1.199-2.883	**0.005**
**Treatment before DLI**			
No treatment/RT	1		
Chemotherapy	1.820	1.009–3.282	**0.046**
Targeted therapy	1.281	0.599–2.738	0.522
**DLI indication**			
AL Relapse	1		
Mixed chimerism	0.434	0.257–0.735	**0.002**
Pre-emptive/prophylaxis	0.431	0.231–0.801	**0.008**
**Number of DLIs**			
Modeled as continuous variable	0.883	0.782–0.997	**0.045**
**Time HCT-first DLI**			
≤180 days	1		
181–356 days	0.765	0.508–1.151	0.199
366–730 days	0.915	0.599–1.395	0.680
>730 days	0.556	0.324–0.955	**0.034**
**Number of transplants**			
1	1		
2	0.682	0.446–1.042	0.077

*AML, acute myeloid leukemia; ALL, acute lymphoblastic leukemia; HLA-id, HLA-identical; MM, mismatched; RT, radiotherapy*.

**Table 4:** Multivariate analysis of overall survival data from first DLI.

**Factor**	**HR**	**95%CI**	***p***
**Age**			
Modeled as continuous variable	1.020	1.008–1.033	**0.001**
**Donor**			
Unrelated	1		
HLA-identical sibling or 1 locus mismatched	1.261	0.854–1.861	0.243
Haploidentical	2.815	1.702–4.656	**0.000**
**DLI indication**			
AL relapse	1		
Mixed chimerism	0.379	0.219–0.646	**0.000**
Pre-emptive/prophylaxis	0.202	0.098–0.415	**0.000**
**Time HCT-first DLI**			
≤180 days	1		
181–356 days	0.741	0.468–1.172	0.201
366–730 days	0.728	0.462–1.147	0.171
>730 days	0.411	0.229–0.740	**0.003**
**Number of DLI**			
Modeled as continuous variable	0.876	0.767–1.000	**0.050**

*HLA-id, HLA-identical; MM, mismatched*.

Since DLIs from haploidentical donor were an independent predictor for worse OS, we compared toxicity and efficacy of DLIs among matched related, unrelated and haploidentical donors. There was no significant difference in the distribution of NRM events among the 3 groups (*p* = 0.313), while acute GVHD was significantly more frequent after DLIs from unrelated donors (21%) and haploidentical donors (28%) in comparison with DLIs from HLA-identical sibling donors (7%) (unrelated DLIs vs. HLA-identical sibling DLIs: *p* = 0.041; haploidentical DLIs vs. HLA-identical sibling DLIs: *p* = 0.020). Moreover, taking in account the 45 patients who received DLIs because of leukemia relapse and were evaluable for response, we observed a significant lower rate of leukemia control (complete remission and stable disease) after DLIs from haploidentical donors (33%) in comparison with DLIs from unrelated donors (78%) (*p* = 0.036), while no significant difference in efficacy was reported if DLIs from matched related donors and those from haploidentical donors were compared (*p* = 0.282).

## Discussion

The first aim of the present survey was to take a picture of the DLI strategy in AL patients in Italian transplant centers. We found that DLIs were administered in 73% of patients after AL clinical recurrence, whereas they represented a way of preventing hematological relapse for less than one third of cases, who received them because of mixed chimerism or MRD positivity. The median time of about 8 months between HCT and first DLI confirmed that immunotherapy was used late in the course of the disease. A few EBMT registry studies have established the efficacy of DLIs either in the setting of overt relapse or used prophylactically. In AML relapse after first HCT, DLIs prolonged OS in comparison with no DLIs (14). Comparison of DLIs and second HCT showed that the clinical benefit of DLIs was comparable to that of salvage HCT (15). Moreover, in a matched-pair analysis, prophylactic DLIs significantly improved outcome in high-risk AML, but failed to achieve an OS advantage in ALL and in standard risk AML (16). In our study, several reasons for reluctance to administer DLI earlier after HCT may be hypothesized. First, physicians may have feared life-threatening complications of DLIs such as GVHD and severe infections. Indeed, in our study, toxicity after DLI was quite low, with fatal adverse events reported in 9% of patients, confirming the NRM incidence reported in previous studies. Moreover, the incidence of severe acute and chronic GVHD was lower than that reported in other registry studies (17), although our analysis may have been limited by the small number of informative patients. Second, a prevention strategy needs standardized markers of MRD and regular monitoring after CT, which might not be available in all Italian centers. Third, contacting and preparing donors can be time-consuming, particularly if they are volunteer donors and lymphocyte donation has to be authorized by a GITMO committee, which is in charge of reviewing clinical HCT history and indication for DLI. Cryopreservation of unmanipulated mobilized PBSC instead of leukapheresis products can enhance DLI availability and accelerate infusions; however, data of the GITMO registry did not allow identification of the two different products.

As expected, we reported a significant OS benefit for patients receiving DLI because of mixed chimerism or MRD positivity in comparison with patients receiving DLI because of hematological relapse. These outcomes are in line with those reported by the EBMT and the Japanese registry studies (14, 15, 18). Moreover, multivariate analysis showed that the greater the number of DLIs administered, the greater the OS improvement. In our study, about 60% of patients received a DLI schedule including more than one DLI at escalating doses, with a median interval of about 1 month between two subsequent infusions. A multiple DLI schedule was administered in a higher percentage of patients compared to previous registry studies, in which 49–61% of patients received one single dose. The more favorable outcome observed in our study for DLIs administered at least 2 years after HCT could reflect the greater clinical benefit of DLIs in late relapses in comparison with early recurrences after HCT, as already reported (14). Multivariate analysis showed no better outcome for patients who received chemotherapy or targeted treatments in association with DLI. These treatments were combined in 57% of patients at the time of first DLI and in a much lower percentage of patients at the following infusions. Although chemotherapy before DLI may theoretically induce leukemia debulking and improve DLI response, no advantage of chemotherapy plus DLI over DLI alone was observed in the AML relapse (18) or pre-emptive settings (19). More promising results were shown by hypomethylating agents: a few cycles of azacitidine or decitabine before DLI in relapsed patients with myeloid neoplasms could activate immune response and promoted some long-term responses, even if the latter were observed in small samples of patients and need confirmation in larger prospective studies (20–23).

In our study, older age of recipients and haploidentical donors were identified as adverse prognostic factors. Although pediatric patients were included in the study, they represented only 5% of the patients, therefore the worse outcome should be probably referred to the elderly adult patients. Moreover, a significantly shorter OS was reported by DLI from mismatched related donors, who included almost exclusively haploidentical donors. The inferior outcome seems to be caused by both lower efficacy in leukemia relapses and more toxicity, in term of acute GVHD, but these results should be interpreted with caution, because of the small number of DLIs from haploidentical donors and the heterogeneity of the GVHD prophylaxis platforms used in our study. Moreover, median dose of the first DLI was 1 log lower after haploidentical transplants in comparison with matched related and unrelated transplants and sequential doses were administered less often after haploidentical than after other HCTs: therefore, inferior doses could have impaired efficacy. Large prospective studies comparing DLIs from haploidentical and conventional donors are still lacking, particularly in the setting of the leukemia relapse. In the context of a prophylactic or pre-emptive strategy, a few small studies comparing T-repleted haploidentical or HLA-identical DLIs in refractory or very high-risk AML observed higher rates of acute GVHD and NRM (24, 25), while a large recent prospective study including 189 AL patients in first complete remission reported a prolonged graft and relapse-free survival after haploidentical HCT with an homogeneous ATG-based prophylaxis followed by DLI in comparison with HCT from matched related donors (26). Clinical trials are needed to establish the optimal timing and cell dose in both therapeutic and prophylactic settings after haploidentical HCT and the relationship with GVHD and disease response (27).

Although the GVL effect has been reported to be lower in ALL than in myeloproliferative diseases (8, 16), in our study, ALL patients had a long-term outcome comparable to that of AML patients. Indeed, at present, other options such bispecific antibodies or chimeric antigen receptor-T cells seem to be more appealing than DLIs for the prevention and treatment of ALL relapse.

DLI administration was followed by a second HCT in 46 patients. It could be hypothesized that the second transplant was performed in patients not achieving a durable complete response after DLI. Therefore, in these patients, DLI represented a “bridge to” a second salvage HCT, allowing them to achieve a slight, but not statistically significant, OS prolongation compared to patients who received DLI alone.

We acknowledge that this study has some limitations. One is the heterogeneity of recipient and donor features of the HCTs included in the study, with 34 participating centers, the majority of which provided data for <10 patients. Another limitation is that only some of the Italian centers agreed to the second phase of the study. Therefore, evaluation of toxicity and clinical response to DLIs was based on a smaller patient population.

However, this survey presents the current “state of the art” of DLI strategy in AL in Italy and allows us to make a few practical and research considerations. From the organizational point of view, the GITMO network may promote a policy of DLI administration as pre-emptive treatment either allowing all centers to detect MRD in AL patients in centralized laboratories or accelerating authorization for leukaphereses from volunteer donors. Moreover, this survey could be the basis for further studies, either retrospective, including more homogeneous populations, or prospective, aiming to address unresolved items, such as DLI from haploidentical donors and DLI schedules according to different indications and different donors.

## Data Availability Statement

The raw data supporting the conclusions of this article will be made available by the authors, without undue reservation.

## Ethics Statement

The study involving human participants was reviewed and approved by the Ethics Committee of the center of the national principal investigator, called Comitato Unico Regionale Friuli-Venezia Giulia on 2017, 3 October (Protocol No. 26522) and by the Ethics Committees of all participating institutions. A written and informed consent was obtained from all patients according to the Declaration of Helsinki.

## Author Contributions

FP, AS, FL, FC, BB, and FB designed the study. EO and SM contributed clinical data from the registry. AP, WA, GS, RS, NM, IC, MM, CB, CM, and RF contributed clinical data from their site. FP, FL, and MI analyzed the data and prepared the manuscript. All authors reviewed and approved the final version of the manuscript.

## Conflict of Interest

The authors declare that the research was conducted in the absence of any commercial or financial relationships that could be construed as a potential conflict of interest.

## Appendix 1: Participant Centers and Principal Investigators

CIC 141, Brescia: M. Malagola

CIC 163, Piacenza: D. Vallisa

CIC 231, Torino: L. Giaccone

CIC 232, Roma, “La Sapienza” University Hospital: A.P. Iori

CIC 240, Bologna: F. Bonifazi

CIC 245, Parma: L. Prezioso

CIC 248, Pescara: P. Olioso

CIC 265, Milano Ospedale Maggiore: F. Onida, G. Saporiti

CIC 286, Pavia: P. Bernasconi

CIC 299, Bolzano: I. Cavattoni

CIC 304, Firenze: A Gozzini

CIC 305, Candiolo: M Aglietta

CIC 307, Roma Università Cattolica SC:P.Chiusolo

CIC 331, Milano Istituto Europeo Oncologico: G. Martinelli

CIC 332, Taranto: G. Pisapia

CIC 354, Milano, Humanitas Cancer Center: L.Castagna

CIC 526, San Giovanni Rotondo (FG): A.M. Carella

CIC 544, Monza: P. Pioltelli

CIC 557, Pavia Clinica Pediatrica: M. Zecca

CIC 587, Reggio Calabria: T. Moscato

CIC 606, Cuneo: R. Sorasio, N. Mordini

CIC 623, Verona: A. Vassanelli

CIC 649, Bari: G. Specchia

CIC 658, Bergamo: A. Rambaldi, C. Micò

CIC 693, Palermo: M. Musso

CIC 705, Udine: F. Patriarca

CIC 756, Roma Tor Vergata: W. Arcese

CIC 788, Ancona: I. Scortechini

CIC 789, Napoli “Cardarelli”: A. Picardi

CIC 796, Roma “Bambin Gesù”: F. Locatelli

CIC 797, Vicenza: C. Borghero

CIC 811, Cagliari: E. Piras

CIC 813, Milano “San Raffaele”: F. Ciceri

CIC 825, Alessandria: M. Pini.
